# Epigenetic reprogramming induced by key metabolite depletion is an evolutionarily ancient path to tumorigenesis

**DOI:** 10.1242/dmm.052313

**Published:** 2025-06-16

**Authors:** Zhe Chen, Xiaomeng Zhang, Mingxi Deng, Chongyang Li, Thi Thuy Nguyen, Min Liu, Kun Dou, Toyotaka Ishibashi, Jiguang Wang, Yan Yan

**Affiliations:** ^1^ Division of Life Science, The Hong Kong University of Science and Technology, Hong Kong 999077, China; ^2^ School of Life Science and Technology, ShanghaiTech University, Shanghai 201210, China; ^3^ School of Life Science, Peking University, Beijing 100871, China; ^4^ Department of Chemical and Biological Engineering, State Key Laboratory of Molecular Neuroscience, The Hong Kong University of Science and Technology, Hong Kong 999077, China; ^5^ SIAT-HKUST Joint Laboratory of Cell Evolution and Digital Health, HKUST Shenzhen-Hong Kong Collaborative Innovation Research Institute, Futian, Shenzhen 518045, China; ^6^ Shenzhen PKU-HKUST Medical Center, Shenzhen 518036, China

**Keywords:** *Drosophila melanogaster*, Invertebrate tumor model, Metabolism, Epigenetics, Acetyl-CoA, S-Adenosyl methionine

## Abstract

Tumor growth is a challenge for multicellular life forms. Contrary to human tumors, which take years to form, tumors in short-living species can arise within days without accumulating multiple mutations, raising the question whether the paths to tumorigenesis in diverse species have any commonalities. In a fly tumor model caused by loss of cell polarity genes, we identified two key metabolic changes: first, systemic depletion of acetyl-CoA leading to a reduction in histone acetylation levels and stochastic silencing of actively transcribed genes; and second, defects in the methionine cycle causing systemic depletion of S-adenosyl methionine, which further reduces histone methylation levels and causes stochastic activation of transposons. Perturbation of the methionine metabolic process inhibits tumor growth. To understand the evolutionary origin of tumorigenesis, we performed comparative studies of fly and human tumors and found that human tumors with metabolic signatures similar to those of fly tumors have a lower mutational load, younger patient age and lower DNA methylation levels. This study indicates that depletion of key metabolites is an evolutionarily ancient driving force for tumorigenesis.

## Teaser

**Summary:** Human tumors with similar metabolic signatures to fly tumors have lower mutational load. Depletion of key metabolites may be an evolutionarily ancient driving force for tumorigenesis.

## INTRODUCTION

Cancer is not a disease that only affects humans. The appearance of tumors seems to coincide with the origin of metazoans. Various tumor types have been documented in organisms ranging from hydra, clams, worms, flies and fish to mammals (Athena Aktipis et al., 2015; Scharrer and Lochhead, 1950). Cancers have even been identified in dinosaur fossil records (Ekhtiari et al., 2020; Rothschild et al., 1998, 1999). Tumors in *Drosophila melanogaster* were first reported in 1918 (Stark, 1918), and they represent one of the best-understood tumor models in species outside mammals (Villegas, 2019). For example, three conserved cell polarity genes – *scribble* (*scrib*), *lethal giant larvae* [*lgl*; also known as *l(2)gl*] and *discs-large* (*dlg*; also known as *dlg1*) – are collectively named as neoplastic tumor suppressor genes (Bilder, 2004; Bilder and Perrimon, 2000; Bilder et al., 2000; Gateff, 1978). When fly larvae are homozygous mutant for *scrib*, *lgl* or *dlg*, their imaginal discs and optical lobes, which are proliferative epithelial organs in larvae, grow into amorphous masses and kill the hosts within days (Figueroa-Clarevega and Bilder, 2015; Gateff, 1978). Even for well-studied fly tumors, it is unclear how closely they resemble human tumors, because these fly tumors reach immortality within days while carrying only one mutation. In contrast, human cancers typically develop over years and acquire multiple mutations to enter the path of carcinogenesis.

Cancer cells are generally thought as being in metabolic states distinct from those of normal cells (De Berardinis and Chandel, 2016; Finley, 2023). For example, most cells can use glucose as an energy source through conserved steps of the oxidation of glucose to pyruvate, the conversion of pyruvate to acetyl groups and the further oxidation of acetyl groups through the citric acid cycle (Nelson and Cox, 2017). Warburg showed that tumor tissue slices produced lactate from glucose even in the presence of ample oxygen, which is now known as a metabolic signature of cancer cells (the Warburg effect) (Finley, 2023; Heiden et al., 2009; Warburg, 1956). Genomics and functional studies have identified several metabolic enzymes frequently mutated in human, which support tumor growth. For example, mutations in isocitrate dehydrogenase genes (*IDH1* and *IDH2*) are frequently observed in gliomas and acute myeloid leukemia (Mardis et al., 2009; Yan et al., 2009). *IDH1* and *IDH2* mutations lead to the accumulation of 2-hydroxygulatrate, which inhibits α-ketoglutarate-dependent dioxygenases and alters the histone and DNA methylation pattern (Chowdhury et al., 2011; Dang et al., 2009; Lu et al., 2012; Xu et al., 2011). Mutations in fumarate hydratase (*FH*) and succinate dehydrogenase (*SDH*), which lead to accumulation of fumarate and succinate, predispose patients to various cancer types (Baysal et al., 2000; Burnichon et al., 2010; Tomlinson et al., 2002; Toro et al., 2003). The accumulation of fumarate and succinate can suppress DNA repair pathway (Sulkowski et al., 2018, 2020) and inhibit phosphatase and tensin homolog (PTEN) through succination (Ge et al., 2022). Phosphoglycerate dehydrogenase (*PHGDH*) is frequently amplified in breast cancer and melanoma samples and increases serine flux to support tumor growth (Locasale et al., 2011; Possemato et al., 2011; Sullivan et al., 2019). Metabolic processes are deeply conserved across species, and it is unknown whether metabolic changes underlying tumorigenesis in different metazoan species share any commonalities.

In this study, we first noticed that a population of Lactate dehydrogenase (Ldh)^+^ cells emerges in fly tumors over time, resembling the Warburg effect observed in mammalian tumors. We further found that fly tumors exhibit major changes in glycolysis, tricarboxylic acid (TCA) cycle and oxidative phosphorylation processes, which lead to systemic depletion of acetyl-CoA. Consequently, we observed reduction in H3K9ac and H3K27ac levels and stochastic silencing of actively transcribed genes in these tumors. Moreover, these fly tumors also exhibit defects in the methionine metabolism process, which leads to systemic depletion of the methyl donor S-adenosyl methionine, reduction in histone methylation levels and de-repression of transposons. We then performed comparative studies between fly and human tumors using metabolic signatures and identified that human tumors with high similarity to fly tumors have a lower mutational load, younger patient age and lower DNA methylation levels.

## RESULTS

### A glycolytic Ldh^+^ cell population arises over time in fly tumors

**Fig. 1.**
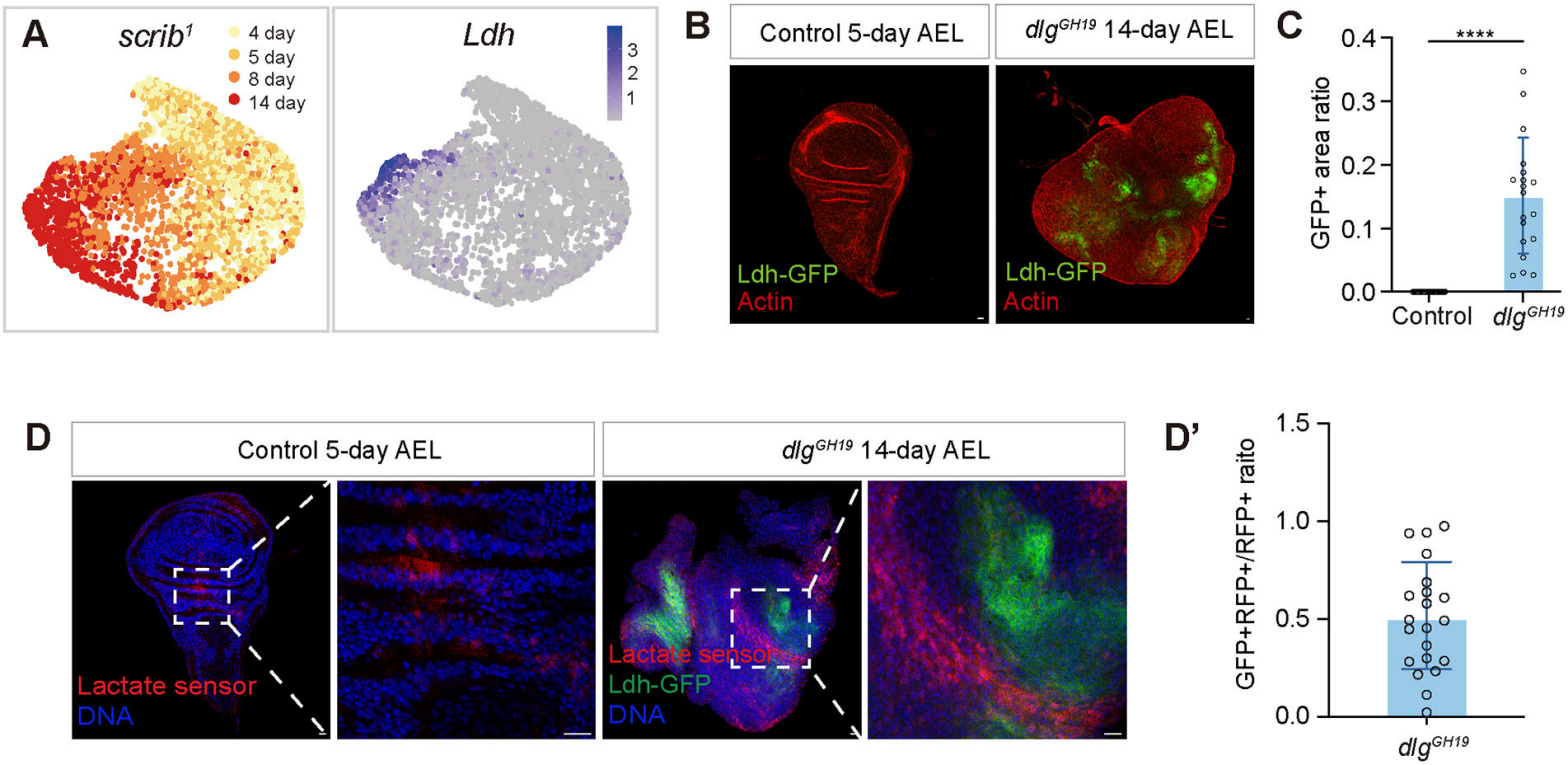
**A Ldh^+^ cell population arises in fly *scrib* and *dlg* mutant tumors over time.** (A) Uniform manifold approximation and projection plot of *scrib* mutant wing disc single cells from different times (left) and expression of the *Ldh* gene (right). (B) Control and *dlg* mutant imaginal discs expressing a Ldh-GFP enhancer trap reporter. AEL, after egg laying. Scale bars: 10 μm. (C) Quantification of Ldh^+^ cell percentage in the control and *dlg* mutant imaginal discs. For B and C, control genotype: *FRT19A; c855aGal4 Ldh-GFP*, 5 days AEL, *n*=25. Experimental group genotype: *dlg^GH19^; c855aGal4 Ldh-GFP*, 14 days AEL, *n*=19. Data represent mean±s.d., with statistical analysis conducted using unpaired two-tailed *t*-test. ****P<0.0001. (D) Control and *dlg* mutant imaginal discs expressing R-iLACCO1, a lactate sensor. Control genotype: *c855aGal4/R-iLACCO1*, 5 days AEL, 24/31, *n*=31. Experimental group genotype: *dlg^GH19^; c855aGal4 Ldh-GFP/R-iLACCO1*, 14 days, 21/21, *n*=21. Scale bars: 10 μm. (D′) Quantification of Ldh^+^ RFP^+^ cell percentage in RFP^+^ cells. Data represent mean±s.d.

The fly tumor models caused by the loss of conserved cell polarity genes, including *scrib*, *dlg* and *lgl*, are among the best-understood tumor models outside of mammals (Bilder, 2004; Bilder and Perrimon, 2000; Bilder et al., 2000). Note that the *scrib* and *dlg* mutants are phenotypically identical, as their encoded proteins function as a complex (Bilder and Perrimon, 2000; Bilder et al., 2000). We used the *scrib* and *dlg* mutant interchangeably in experiments, and the choice of which mutant to use was based on the ease of genetic combination. We have previously used single-cell transcriptomics to profile the fly *scrib* mutant wing disc tumors as they grew from 4 days to 14 days (Deng et al., 2019; Ji et al., 2019). From this dataset, we noticed that a population of Ldh^+^ cells emerged in the late-stage *scrib* mutant tumors (Fig. 1A). We further validated the existence of this Ldh^+^ cell population by examining the expression of a Ldh-GFP enhancer trap line (Wang et al., 2016) in the *dlg* mutant (Fig. 1B,C). Ldh^+^ cell populations were previously shown to be induced in fly wing disc tumors caused by active Pvr (Wang et al., 2016) or Hipk (Wong et al., 2019) signals. Similar to what has been shown in these contexts, the upregulation of the Ldh^+^ cell population in the *scrib/dlg* mutant tumors was also regulated by HIF-1 (also known as HIF) and PI3K/Akt signals (Fig. S1). Specifically, depletion of HIF-1 through RNA interference (RNAi) blocked the increase in Ldh^+^ cells (Fig. S1A), and activation of PI3K/Akt signaling through *Pten* RNAi promoted the expansion of Ldh^+^ cells (Fig. S1B).

We further examined whether the expansion of a *Ldh^+^* glycolytic cell population led to elevation of lactate levels in *dlg* mutant tumors using a biosensor for L-lactate, R-iLACCO1 (Nasu et al., 2023). In the control wild-type wing discs, we observed weak fluorescence level at the hinge region (Fig. 1D). In the 14-day *dlg* mutant tumors, we observed bright red fluorescence signals (Fig. 1D). Notably, the R-iLACCO1 signal was frequently observed in cells adjacent to the Ldh-GFP^+^ cells. On average, only half of the R-iLACCO1^+^ cells were also positively expressing Ldh (Fig. 1D′), indicative of possible lactate transfer among these cells (Brooks, 2018). Together, these data suggest that the *scrib* and *dlg* mutant tumors exhibit characteristics of the Warburg effect as these tumors grow over time.

### Depletion of acetyl-CoA leads to a reduction in H3K9ac and H3K27ac levels and stochastic silencing of actively transcribed genes in fly tumors

**Fig. 2.**
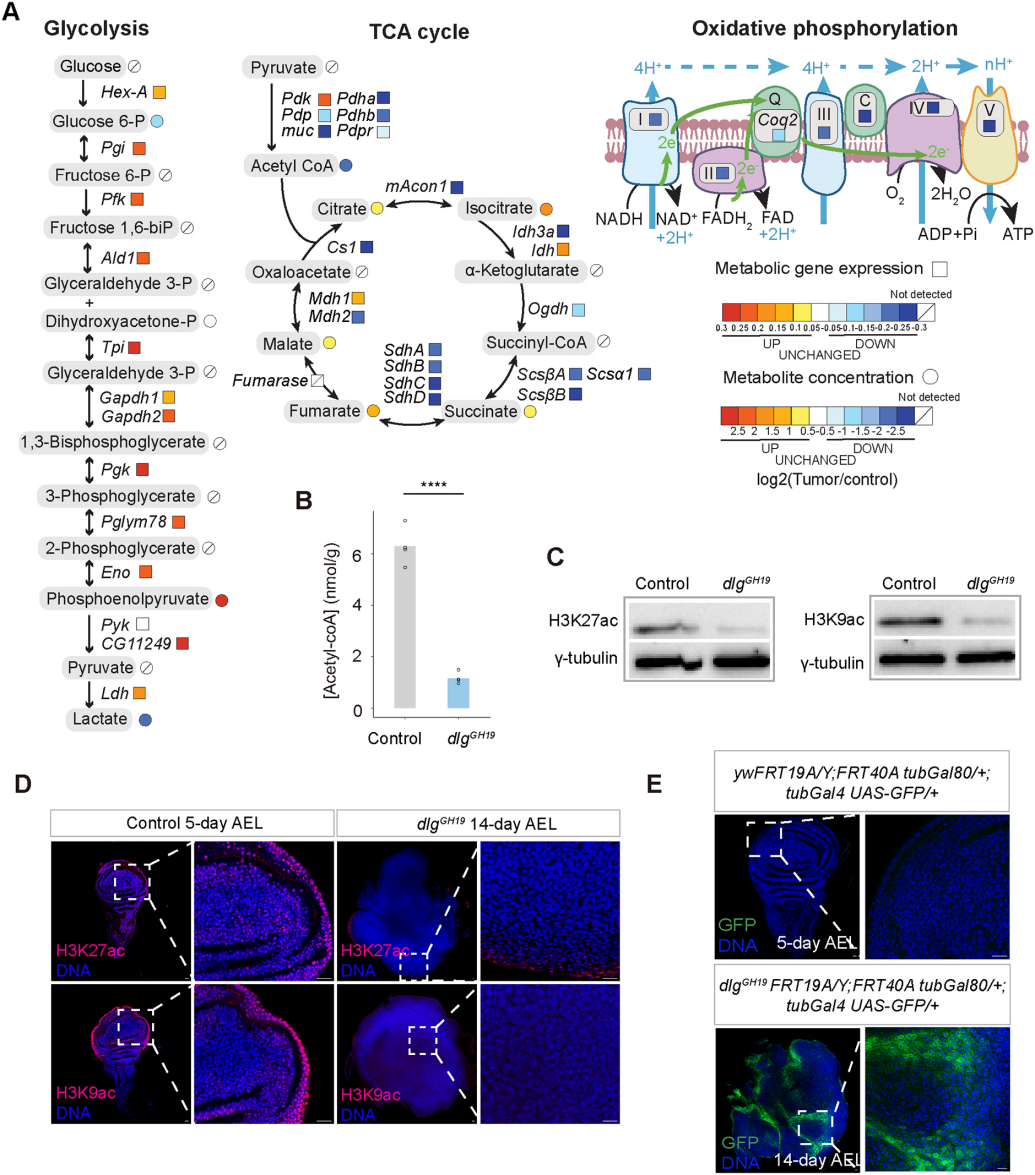
**Systemic depletion of acetyl-CoA leads to a reduction in histone acetylation levels and stochastic silencing of actively transcribed genes in *dlg* mutant tumors.** (A) Plot of metabolic gene expression changes from the *scrib* mutant tumor time-series transcriptomics data (color coded in squares) and metabolite concentration changes between the 13-day *dlg* mutant larvae and control larvae (color coded in circles) for glycolysis, the tricarboxylic acid (TCA) cycle and oxidative phosphorylation. The gene expression change slope value and metabolite concentration fold change value are color coded. (B) Plot of acetyl-CoA concentration in the control and *dlg* mutant larvae. Control group genotype: *FRT19A*, 5 days AEL. Experimental group genotype: *dlg^GH19^*, 13 days AEL. Four repeats per group. Data represent mean values with individual data points overlaid. Statistical analysis was performed using the Wilcoxon rank sum test. ****P<0.0001. (C) Western blot of H3K27ac and H3K9ac from the control imaginal discs and *dlg* mutant tumors. (D) Control and *dlg* mutant imaginal discs stained for H3K27ac and H3K9ac. Control genotype: *FRT19A*, 5 days AEL. Experimental group genotype: *dlg^GH19^*, 14 days AEL. H3K27ac, control, *n*=18, *dlg^GH19^*, *n*=20; H3K9ac, control, *n*=22, *dlg^GH19^*, *n*=15; Scale bars: 10 μm. (E) Control and *dlg* mutant imaginal discs expressing a *tubGal80; tubGal4 UAS-GFP* reporter. Detection of GFP^+^ cells is indicative of loss of tubGal80 expression. Control genotype: *yw*FRT19A/Y; FRT40A tubGal80/+; tubGal4 UAS-GFP/+, *n*=21, 5 days AEL. Experimental group genotype: *dlg^GH19^*FRT19A/Y; FRT40A tubGal80/+; tubGal4 UAS-GFP/+, *n*=33, 32/33, 14 days AEL. Scale bars: 10 μm.

Glycolysis, the citric acid cycle (TCA cycle) and oxidative phosphorylation are tightly coupled processes for glucose breakdown and ATP production (Fig. 2A). In addition to the increase in Ldh^+^ cells in these tumors, we further noticed that genes responsible for the breakdown of glucose to pyruvate were collectively upregulated over time in the *scrib* mutant tumors (Fig. 2A; Fig. S2A). The pyruvate dehydrogenase complex genes, which convert pyruvate to acetyl-CoA for entry into the TCA cycle, were collectively downregulated over time in the *scrib* mutant tumors (Fig. 2A; Fig. S2B). Concurrently, TCA cycle genes were downregulated over time in the *scrib* mutant tumors (Fig. 2A; Fig. S2C). Moreover, genes encoding components of mitochondrial respiratory chain complexes I-V were collectively downregulated over time in the *scrib* mutant tumors (Fig. 2A; Fig. S2D). These data suggest that the breakdown of glucose through glycolysis, the TCA cycle and oxidative phosphorylation to generate ATP likely slows down at the step of pyruvate-to-acetyl-CoA conversion for entry into the TCA cycle in late-stage *scrib* mutant tumors.

We then compared the concentrations of metabolites between the *dlg* mutant larvae and control larvae through metabolomics profiling (Fig. 2A; Table S1). We observed elevated levels of metabolites in the TCA cycle – including citrate, cis-aconitate, isocitrate, succinate, fumarate and malate – in the *dlg* mutant larvae (Fig. 2A), likely indicative of an overall slowdown of metabolite flow through the TCA cycle. Notably, in human cancers caused by mutations in *IDH1*, *FH* or *SDH*, the accumulation of metabolites in the TCA cycle, such as succinate and fumarate, is also frequently observed (Chowdhury et al., 2011; Dang et al., 2009; Ge et al., 2022; Sulkowski et al., 2020; Xu et al., 2011). In fly brain tumors caused by the loss of Brat protein, the accumulation of TCA intermediates was also observed (Bonnay et al., 2020), highlighting that the accumulation of TCA intermediates might represent a highly conserved feature of tumors from different animal species.

Consistent with the downregulation of expression levels of PDH complex genes, which function to convert pyruvate to acetyl-CoA for entry into the TCA cycle, we observed a reduction in acetyl-CoA levels in the *dlg* mutant larvae (Fig. 2B). Acetyl-CoA not only serves as a metabolic intermediate for TCA cycle and lipid synthesis but also acts as the acetyl donor for protein acetylation, particularly histone acetylation (Diehl and Muir, 2020; Pietrocola et al., 2015; Shi and Tu, 2015). We then examined whether depletion of acetyl-CoA in the *dlg* mutant larvae affects protein acetylation levels, in particular, histone acetylation levels. Histone post-translational modifications H3K27ac and H3K9ac are classic markers for actively transcribed chromatin (Nègre et al., 2011; modENCODE Consortium et al., 2010; Tie et al., 2009, 2014). We observed that H3K27ac and H3K9ac levels were much lower in *dlg* mutant wing disc tumors than in wild-type wing discs (Fig. 2C,D). Removal of histone acetylation markers can potentially lead to the silencing of actively transcribed genes (Gallinari et al., 2007). To examine the consequences of an overall reduction in histone acetylation levels in *dlg* mutant tumors, we adopted a *tubGal80-tubGal4* reporter system originally used to monitor loss of heterozygosity in aging intestinal stem cells (Siudeja et al., 2015). In this system, the inactivation of a *tubulin* (*tub*) promoter-driven Gal80 allows the expression of GFP activated by *tub* promoter-driven Gal4 (Siudeja et al., 2015). The inactivation of tubGal80 can result from either DNA mutations or deletions, or from epigenetic silencing in the *tubGal80* locus (Siudeja et al., 2015). Using this system, we found that GFP^+^ cells were never observed in the control imaginal discs, indicating that the *tubGal80-tubGal4* reporter system is not leaky (Fig. 2E). However, we frequently observed GFP^+^ clones in the 14-day *dlg* mutant tumors (Fig. 2E). We have previously performed whole-genome sequencing of *dlg* mutant tumors and did not detect further mutations except the original *dlg* mutation (Ji et al., 2019). Therefore, the stochastic loss of Gal80 likely results from the epigenetic silencing of the *tubGal80* locus in association with the loss of histone acetylation markers.

### Depletion of S-adenosyl methionine leads to a reduction in H3K9me3 levels and transposon activation in fly tumors

**Fig. 3.**
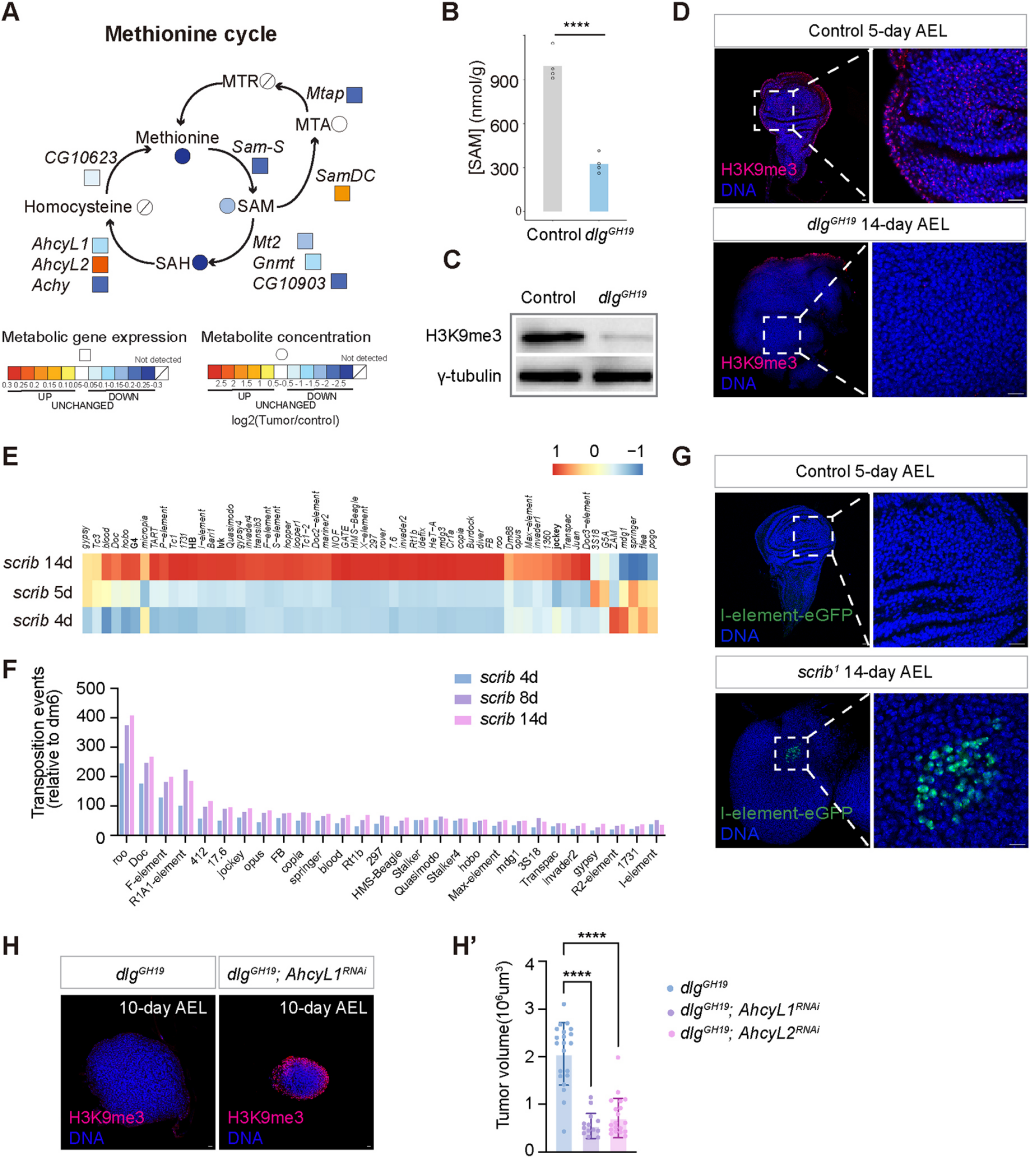
**Systemic depletion of S-adenosyl methionine leads to a reduction in H3K9me3 levels and transposon activation in fly tumors.** (A) Plot of gene expression changes from the *scrib* mutant tumor time-series transcriptomics data (color coded in squares) and metabolite concentration changes between the 13-day *dlg* mutant larvae and control larvae (color coded in circles) in the methionine cycle. (B) Plot of S-adenosyl methionine (SAM) concentration in the control and *dlg* mutant larvae. Control group genotype: *FRT19A*, 5 days AEL. Experimental group genotype: *dlg^GH19^*, 13 days AEL. Four repeats per group. Data represent mean values with individual data points overlaid. Statistical analysis was performed using the Wilcoxon rank sum test. ****P<0.0001. (C) Western blot of H3K9me3 from control and *dlg* mutant imaginal discs. (D) Control and *dlg* mutant imaginal discs stained for H3K9me3. Control genotype: *FRT19A*, 5 days AEL, *n*=21. Experimental group genotype: *dlg^GH19^*, 14 days AEL, *n*=16. Scale bars: 10 μm. (E) Heatmap of transposon expression data from the 4-day, 5-day and 14-day AEL *scrib* mutant wing imaginal discs. (F) Bar plot of transposition events mapped in the 4-day, 8-day and 14-day AEL *scrib* mutant wing imaginal discs. (G) Control and *scrib* mutant imaginal discs expressing an I-element-eGFP reporter. Control genotype: I-element-eGFP; FRT82B, *n*=23, 5 days AEL. Experimental group genotype: I-element-eGFP; *scrib^1^* FRT82B, *n*=29, 13/29, 14 days AEL. Scale bars: 10 μm. (H) Imaginal discs of stained for H3K9me3 (red) and DNA (blue). Scale bars: 10 μm. (H′) Quantification of tumor sizes for the *dlg* mutant tumors in the control, *AhcyL1^RNAi^* and *AhcyL2^RNAi^* groups. Control genotype: *dlg^GH19^; c855aGal4/+*, 10 days AEL, *n*=21. Experimental group genotypes: *dlg^GH19^; c855aGal4/AhcyL1^RNAi^*, 10 days AEL, *n*=15. *dlg^GH19^; AhcyL2^RNAi^/+; c855aGal4/+*, 10 days AEL, *n*=21. Data represent mean±s.d., with statistical analysis conducted using one-way ANOVA. ****P<0.0001.

We next examined additional metabolic changes observed in the *dlg* mutant larvae in comparison with the wild-type larvae and noticed a significant reduction in methionine cycle-related metabolites, such as S-adenosyl methionine, in the *dlg* mutant larvae (Fig. 3A,B). S-Adenosyl methionine is a major methyl donor. Therefore, we examined whether histone methylation status was affected in the *dlg* mutant tumors. We found that the level of H3K9me3, a well-recognized repressive chromatin marker (Nègre et al., 2011; modENCODE Consortium et al., 2010), was significantly reduced in the *dlg* mutant tumors in comparison with the control (Fig. 3C,D).

Removal of silencing markers such as H3K9me3 can potentially lead to transposon activation and loss of gene silencing in the heterochromatin region (Wood et al., 2016). We examined the transcript levels of multiple transposons in the *scrib* mutant tumors and observed de-repression of many transposons in the 14-day *scrib* mutant tumors (Fig. 3E; Table S2). Consistently, we performed whole-genome sequencing for the 4-day, 8-day and 14-day *scrib* mutant tumors, mapped transposition events and noticed that transposition events were more frequent in 8-day and 14-day mutant tumors in comparison with those in 4-day mutant tumors (Fig. 3F; Table S3). We then adopted a reporter for transposon activation, I-element-eGFP reporter, which only produces GFP signal upon retrotransposition (Wang et al., 2018). In the control wing imaginal discs, we never observed any GFP signal, indicative of tight repression of transposons in the wild-type somatic cells (Fig. 3G). In the 14-day *scrib* mutant tumors, we observed GFP^+^ cells in ∼50% of samples we examined (Fig. 3G), indicating that the reporter was not only reverse transcribed but its cDNA often successfully landed back into the genome (Wang et al., 2018). Together, these results suggest that the late-stage *scrib/dlg* mutant tumors likely harbor an increasingly diverse cell population with stochastic transposon activation. To determine whether depletion of S-adenosyl methionine is a driving force for tumorigenesis, we examined the functional consequences of perturbing enzymes in the methionine cycle in the *dlg* mutant tumors. Interestingly, we found that knockdown of *AhcyL1* and *AhcyL2*, which potentially elevates S-adenosyl homocysteine hydrolase Ahcy activity (Parkhitko et al., 2016), elevated H3K9me3 levels and strongly inhibited *dlg* mutant tumor growth (Fig. 3H). Note that perturbation of AhcyL1 and AhcyL2 activity in normal wing discs did not affect normal developmental growth (Fig. S3).

### Identification of an evolutionarily ancient category of human tumor samples that metabolically resemble fly tumors

**Fig. 4.**
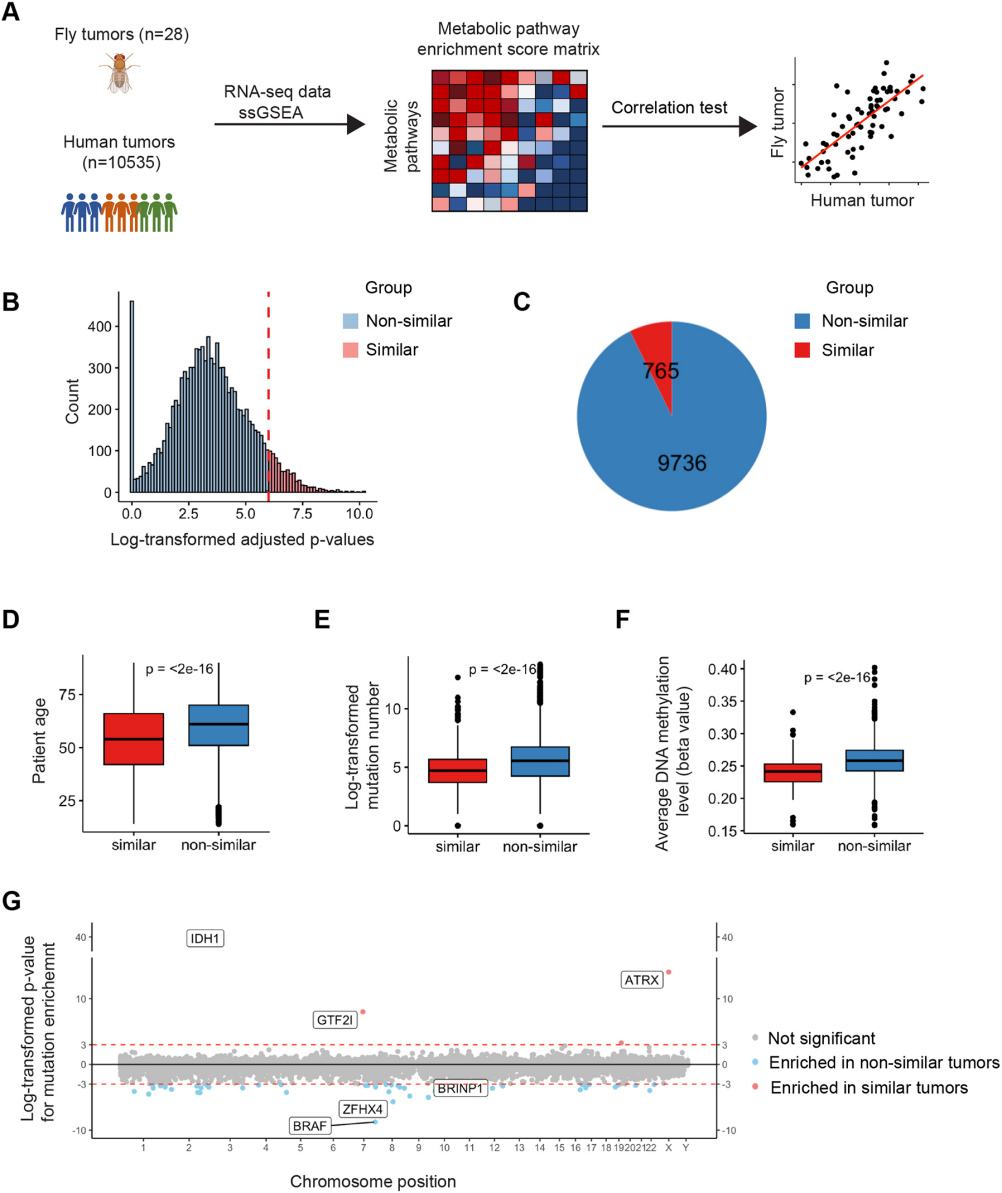
**The metabolic gene expression signatures of fly and human tumors.** (A) Schematic view of using gene expression data from fly and human tumor samples to identify human tumor samples that metabolically resemble fly tumors. RNA-seq, RNA-sequencing; ssGSEA, single-sample gene-set enrichment analysis. (B) Plot of the distribution of adjusted *P*-values for metabolic similarity tests between human tumor samples and the fly *scrib* mutant tumors. *P*-values are calculated by Pearson correlation test. Human tumor samples with *P*-values lower than 1×10^−6^ (colored in red) are recognized as samples with significantly similar metabolic patterns to fly tumors. (C) 765 out of 10,501 human tumor samples exhibit metabolic signatures similar to those of fly tumors based on *P*-values of Pearson correlation test. (D) Patients with tumors metabolically similar to the fly tumors are significantly younger than others. Data represent distribution of patient age of two human tumor groups, and *P*-value is calculated by unpaired two-tailed *t*-test. (E) Human tumor samples metabolically similar to the fly tumors acquire a significantly lower number of mutations than other samples. Data represent log2-transformed count number of mutation events detected in two human tumor groups, and *P*-value is calculated by unpaired two-tailed *t*-test. (F) Human tumor samples metabolically similar to the fly tumors show a lower level of DNA methylation than other samples. Data show average beta-values, which represent the DNA methylation level of two human tumor groups, and *P*-value is calculated by unpaired two-tailed *t*-test. (G) Mutation landscape of human tumor samples metabolically similar to the fly tumors. Dots in the upper panel represent mutation events, which are significantly enriched in the metabolically similar tumor group, while dots in the lower panel are events enriched in the non-similar tumor group. *P*-values in the *y*-axis are calculated by Fisher's exact test, which evaluates whether one mutation event is correlated with tumor metabolic pattern. Dots with *P*-values lower than 1×10^−3^ are colored in red/blue and represent mutation events that are significantly enriched or lost in the metabolically similar tumor group.

Major metabolic pathways are highly conserved across animal species. Using Kyoto Encyclopedia of Genes and Genomes (KEGG) and Metabolic Atlas as references, we annotated 72 metabolic pathways conserved between fly and human (Table S4). We then use transcriptomics data from the *scrib* mutant tumor samples (Ji et al., 2019) and 10,501 human tumor samples in The Cancer Genome Atlas (TCGA) database to perform single-sample gene-set enrichment analysis (ssGSEA) for the 72 conserved metabolic pathways (Fig. 4A). Using metabolic pathway enrichment scores as a matrix, we further performed correlation analysis to identify human tumor samples with high metabolic similarity to fly tumors (Fig. 4A). In the top 7% human tumors with high metabolic similarity to fly tumors (Fig. 4B,C) (*n*=765/10,501), we identified younger patient age (Fig. 4D), a lower mutational load (Fig. 4E) and a lower level of DNA methylation (Fig. 4F), in comparison with tumor samples classified as non-similar to fly tumors. The human tumor samples that metabolically resemble fly tumors showed strong enrichment for mutations in *IDH1*, *GTF2I* and *ATRX* (Fig. 4G). *GTF2I* encodes a transcriptional factor with high mutation frequency observed in thymoma (Petrini et al., 2014). *ATRX* is a chromatin remodeler with high mutation frequency in glioma (Koschmann et al., 2016). Consistently, the tumor samples with high similarity to fly tumors were highly enriched in eight cancer types out of the total 33 cancer types, including B-cell lymphoma, glioblastoma, glioma, ovarian adenocarcinoma, testicular germ cell tumor, thymoma and uterine carcinosarcoma (Fig. S4). For the eight cancer types with a high percentage of tumors metabolically similar to fly tumors, the patient survival curves did not show significant differences between the two groups, except for glioma, for which patients with tumors metabolically similar to fly tumors had better survival outcomes (Fig. S5).

To further validate these results, we obtained an independent cohort of 185 Chinese Glioma Genome Atlas (CGGA) glioma tumor data and separated them into two groups based on similarity of their metabolic signature to that of fly tumors (Fig. S6A,B). Similarly, for this independent dataset, the patients in the similar group were significantly younger than those in the non-similar group (Fig. S6C). The human glioma samples that metabolically resembled fly tumors harbored a significantly lower number of mutations (Fig. S6D) and showed strong enrichment for *IDH1* mutations (Fig. S6E), and the patients in the similar group had better survival outcome (Fig. S6F).

Together, these data show that human tumor samples sharing similar metabolic signatures with fly tumors harbor fewer mutations, which suggests that they may be less dependent on mutation accumulation over time for malignant growth.

## DISCUSSION

Here, we demonstrated that epigenetic reprogramming induced by key metabolite depletion is an evolutionarily ancient driving force for immortal growth, lowering the barrier to tumorigenesis and the need for mutation accumulation over time. Interestingly, a recent study also demonstrated that the transient silencing of Polycomb complex is sufficient to drive malignant tumor formation in *Drosophila* (Parreno et al., 2024). Notably, even in human cancers, ∼5.3% of tumors do not harbor any known cancer-driver mutations (ICGC/TCGA Pan-Cancer Analysis of Whole Genomes Consortium, 2020). For example, mutations are rarely found in childhood brain tumors (Mack et al., 2014). Therefore, epigenetic changes induced by metabolic stress may represent an evolutionarily ancient route to malignant growth, which co-exist with the acquisition of multiple mutations in long-living species. For the portion of evolutionarily ancient human tumors, it will be interesting to use fly tumors as a model to test therapeutic interventions. For example, we have found that inhibition of AhcyL1 and AhcyL2, enzymes functioning in the methionine metabolic process, potently inhibits fly tumor growth. In the future, it would be interesting to test whether methionine metabolic intervention benefits patients with evolutionarily ancient tumors.

The metabolic network is largely conserved among animal species. The accumulation of TCA intermediates is observed in both human and fly tumors. Although many studies have focused on the accumulation of oncometabolites (Liu and Yang, 2021), our work here highlights the consequences of depletion of metabolites, including acetyl-CoA and S-adenosyl methionine, which are key substrates for histone post-translational modifications. Notably, we only examined a few general histone acetylation and methylation markers and observed a global reduction in these tumors. Whether specific histone modifications or specific gene loci are resistant to the depletion of metabolites is unclear at this stage. Similar to the concept of oncogenes and tumor suppressor genes, the depletion of metabolites is likely as important as the accumulation of oncometabolites in tumorigenesis processes. A global reduction in histone acetylation and methylation levels can lead to stochastic gene silencing and activation, the implications of which need to be further explored in human tumors.

## MATERIALS AND METHODS

### *Drosophila* genetics and stocks

*Drosophila melanogaster* stocks were maintained at room temperature, and fly crosses were raised in a 25°C fly incubator. The fly strains used in this study were as follows: *scrib^1^* FRT82B/TM6B (Bilder and Perrimon, 2000), *dlg^GH19^* FRT19A/FM7C (Ji et al., 2019), *c855aGal4* [Bloomington *Drosophila* Stock Center (BDSC 6990)], *Ldh-GFP* (Wang et al., 2016), R-iLACCO1 (Nasu et al., 2023), I-element*-*eGFP-reporter (Wang et al., 2018), *FRT40A tubGal80; tubGal4 UAS-GFP* (a gift from the Xi laboratory, National Institute of Biological Sciences, Beijing, China), *HIF-1* RNAi (BDSC 26207), *Pten* RNAi [Vienna *Drosophila* Resource Center (VDRC) 101475], *AhcyL1* RNAi (BDSC 28523), *AhcyL2* RNAi (BDSC 61913) and control strains (OreR or w1118).

### Wing discs dissection and immunostaining

Wing discs and tumors were dissected, fixed and stained following standard protocols. The primary antibodies used in this study were rabbit anti-H3K9ac (Abcam, ab4441, 1:1000), rabbit anti-H3K27ac (Abcam, ab4729, 1:1000) and rabbit anti-H3K9me3 (Abcam, ab8898, 1:1000). Secondary antibodies used were Goat anti-Rabbit IgG (H+L) Secondary Antibody, Alexa Fluor™ 546 (Invitrogen, A11035, 1:1000) and Goat anti-Mouse IgG (H+L) Secondary Antibody, Alexa Fluor™ 546 (Invitrogen, A11030, 1:1000). Hoechst 33342 (Invitrogen, H3570, 1:10,000) and Alexa Fluor™ 647 Phalloidin (Invitrogen, A22287,1:1000) were used to stain DNA and actin for cell outline, respectively.

### Western blotting

Forty pairs of wing imaginal discs or tumors were dissected in PBS. Tissue samples were vortexed in RIPA lysis buffer for 1 min, followed by sonication. The sonication program settings included an amplitude of 50, a processing time of 15 min, a pulse-on time of 15 s and a pulse-off time of 10 s. After cell lysis, the samples were centrifuged at 18,400 ***g*** for 10 min. The supernatant was then measured for protein concentration using a Pierce BCA Protein Assay Kit. The primary antibodies used were rabbit anti-H3K9ac (Abcam, ab4441, 1:1000), rabbit anti-H3K9me3 (Abcam, ab8898, 1:1000), rabbit anti-H3K27ac (Abcam, ab4729, 1:1000) and mouse anti-gamma-tubulin (Abcam, ab27074, 1:1000).

### Confocal imaging and data analysis

Images were acquired on a Leica TCS SP8 confocal microscope. Wing discs and tumors were taken as *z*-stacks for volume quantification using ImageJ and CellProfiler (Stirling et al., 2021).

### Metabolomics

Embryos within a 3-h window were collected from apple juice plates and aged to larvae of appropriate ages. Wild-type larvae [5 days after egg laying (AEL)] and *dlg* mutant larvae (13 days AEL) (12 larvae for each technical replicate, *n*=4 for each genotype) were sent as frozen samples to Biotree for 600MRM metabolite analysis with liquid chromatography–mass spectrometry. Metabolite concentrations (*C*_M_; nmol/g) were calculated using the following formula:
(1)

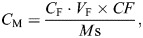
where *C*_F_ denotes the final concentration (μmol/l) and is a product of the calculated concentration (μmol/l) detected by the machine and dilution factor; *V*_F_ (μl) represents the volume of the sample; *CF* is the dilution factor in sample preparation; and *M*_s_ (mg) is the mass of the sample. The statistical significance of differences between two genotypes was calculated using Wilcoxon rank sum test.

### Metabolic signature analysis based on transcriptomics data

Bulk and single-cell RNA-sequencing data on *scrib* mutant tumors were obtained from our previous publications (Deng et al., 2019; Ji et al., 2019) (Gene Expression Omnibus accession numbers GSE130243 and GSE130566). The gene expression data for 10,501 tumors of 33 cancer types were obtained from TCGA Data Portal. The metabolic pathway annotation tables were manually curated from the KEGG database, the Human-GEM and the Fruitly-GEM Metabolic Atlas (Wang et al., 2021).

For a given tumor sample, an enrichment score for a specific metabolic pathway was calculated through ssGSEA based on gene expression data. The similarity between a given human tumor sample and fly *scrib* mutant tumor was then calculated by Pearson correlation test using the enrichment scores of 72 conserved metabolic pathways. We set a cutoff on adjusted *P*-values of correlation coefficients to classify human tumors into metabolically similar and non-similar groups. We then used available somatic mutation information from TCGA and applied Fisher's exact test to examine significantly enriched or lost mutation events in human tumors metabolically similar to fly tumors in comparison with human tumors metabolically non-similar to fly tumors. The processed pan-cancer expression data and somatic mutation information of TCGA were provided by University of California, Santa Cruz Xena. To verify our findings using the TCGA dataset, we applied the same methods on the expression and mutation data of glioma samples from CGGA. The gene expression data and somatic mutation information of CGGA are publicly available at the CGGA website.

For bulk RNA-sequencing analysis, the transcript abundance (as denoted in fragments per kilobase per million reads) of transposons was counted by using piPipes as described in Han et al. (2015). Gene expression heatmaps were generated using the pheatmap package, and principal component analysis was performed using FactoMineR and ggplot2 packages. Single-cell data merging and gene expression plot generation were performed using Seurat package (Hao et al., 2024).

### Whole-genome sequencing and transposition events mapping

The *scrib* mutant tumors of different stages were dissected in PBS. Genomic DNA was extracted using a DNeasy Blood and Tissue Kit (Qiagen, 69504). Paired-end sequencing was performed on an Illumina^®^ sequencing platform, with a read length of 150 bp at each end. We first mapped raw whole-genome sequencing data to the dm6 genome to estimate mappability via BWA v0.7.17 and randomly selected sequencing reads of each library by seqtk to obtain the same genome coverage for comparison. The transposition events of each sample were calculated by TEMP (Zhuang et al., 2014).

## Supplementary Material

10.1242/dmm.052313_sup1 Supplementary information

Table S1. List of metabolite concentrations from the control and the *dlg* mutant larvae as measured in the 600MRM metabolite analysis with LC-MS.

Table S2. The expression of transposons in the *scrib* mutant tumors over time.

Table S3. The transposition events mapped in the *scrib* mutant tumors over time in comparison with the reference genome.

Table S4. List of conserved metabolic genes and pathways in *Drosophila Melanogaster* and human.
