# Supplementary material for: Epigenetic reprogramming induced by key metabolite depletion is an evolutionarily ancient path to tumorigenesis
Source: Dis Model Mech. 2025 Jun 16;18(6):dmm052313. doi: 10.1242/dmm.052313 (PMC12208194; doi:10.1242/dmm.052313)
Supplement: Supplementary information [file dmm-18-052313-s1.pdf]

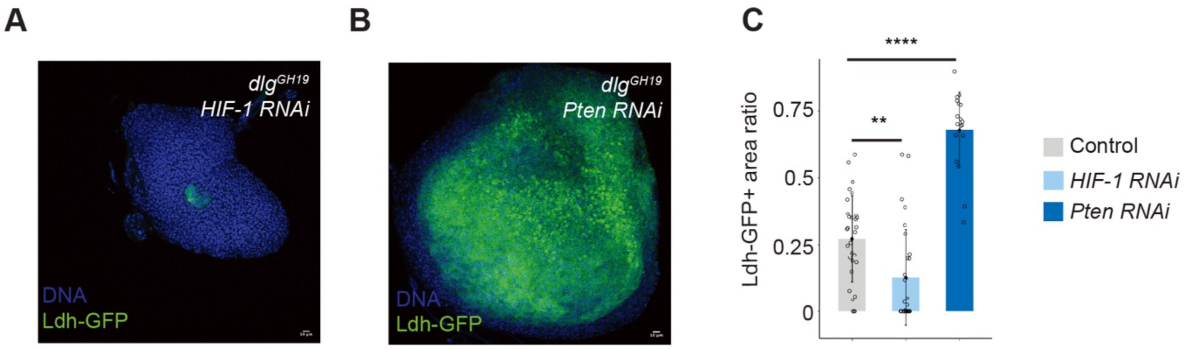

Fig. S1. The rise of Ldh+ cell population in the *dlg* mutant tumors is regulated by HIF-1 and Pten.

(A) The *dlg* mutant tumors expressing a Ldh-GFP reporter and a *HIF-1* RNAi construct. Scale bar: 10μm.

(B) The *dlg* mutant tumors expressing a Ldh-GFP reporter and a *Pten* RNAi construct. Scale bar: 10μm.

(C) Quantification of Ldh-GFP+ cell ratio in the *dlg* mutant tumors when *HIF-1* RNAi or *Pten* RNAi is expressed in comparison with control. Control, n=30; *HIF-1* RNAi, n=29; *Pten* RNAi, n=19. Data represent mean ± SD, with individual data points overlaid. Statistical analysis was performed using the Wilcoxon Rank Sum test. **, $P < 0.01$; ****, $P < 0.0001$.

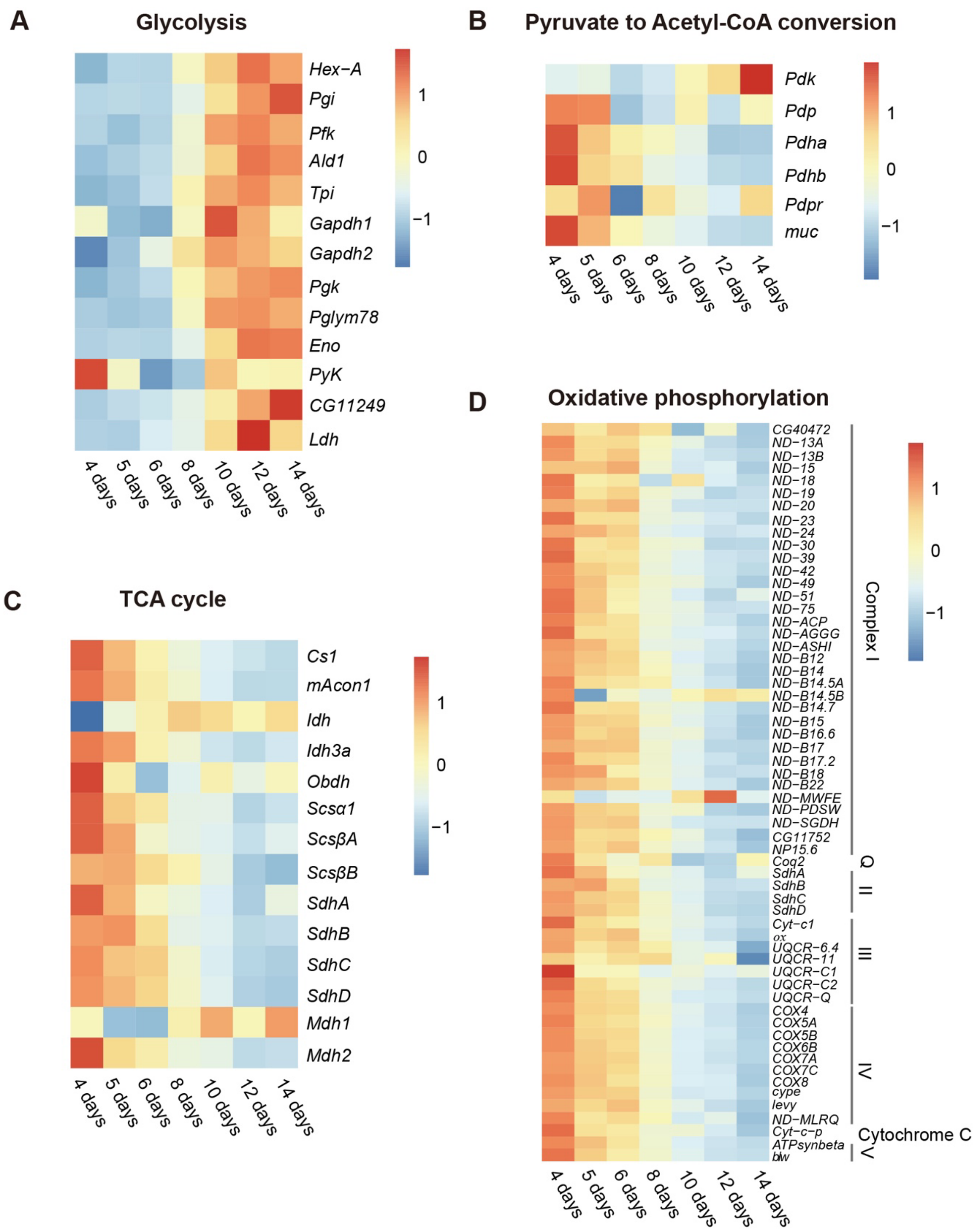

Fig. S2. Heatmap plot of the expression of glucose metabolic pathway gene sets in the *scrib* mutant tumors over time.

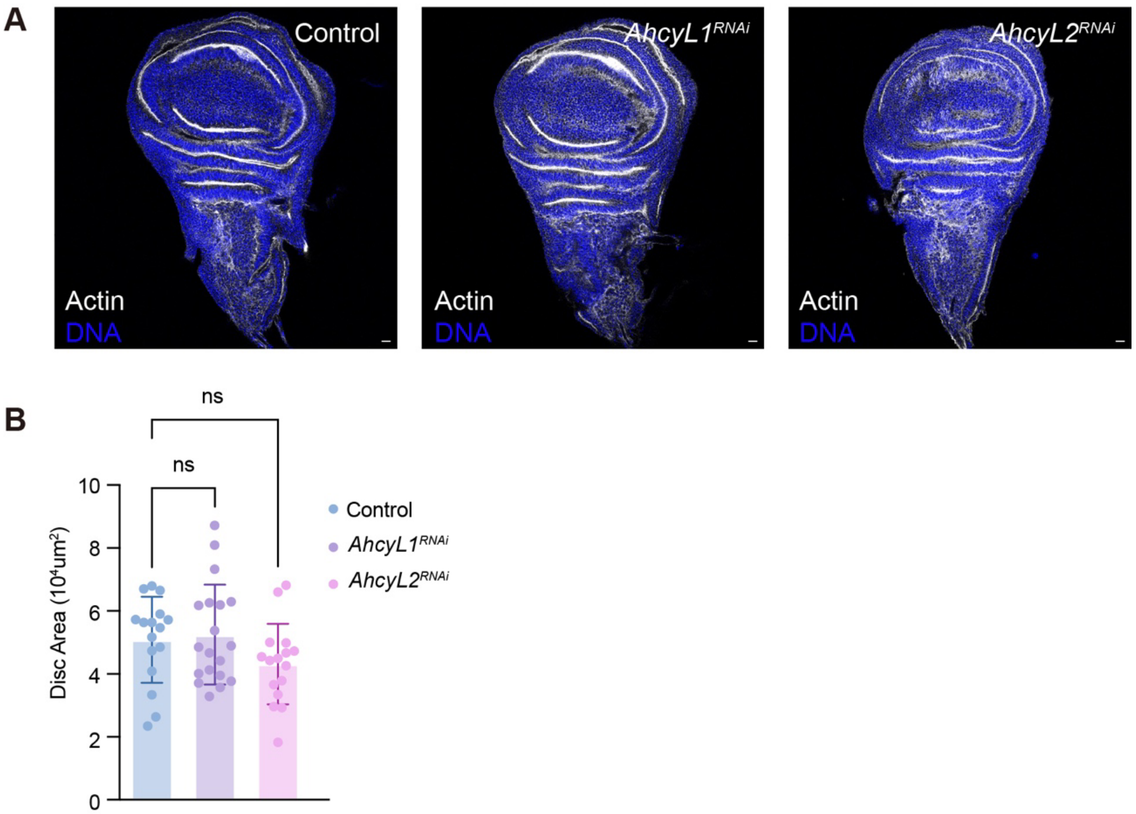

Fig. S3. Perturbation of AhcyL1 and AhcyL2 does not affect normal wing imaginal disc growth.

(A) Wing imaginal discs stained for actin (gray) and DNA (blue). Scale bar: 10µm.

(B) Quantification of wing imaginal disc size. For (A-B), control genotype: c855aGal4/+, n=16; Experimental group genotypes: *c855aGal4/AhcyL1^{RNAi}, n=19; AhcyL2^{RNAi}/+; c855aGal4/+, n=16.* Data represent mean ± SD, with statistical analysis conducted using one-way ANOVA. ns, P =0.1429; ns, P=0.5666.

Disease Models & Mechanisms • Supplementary information

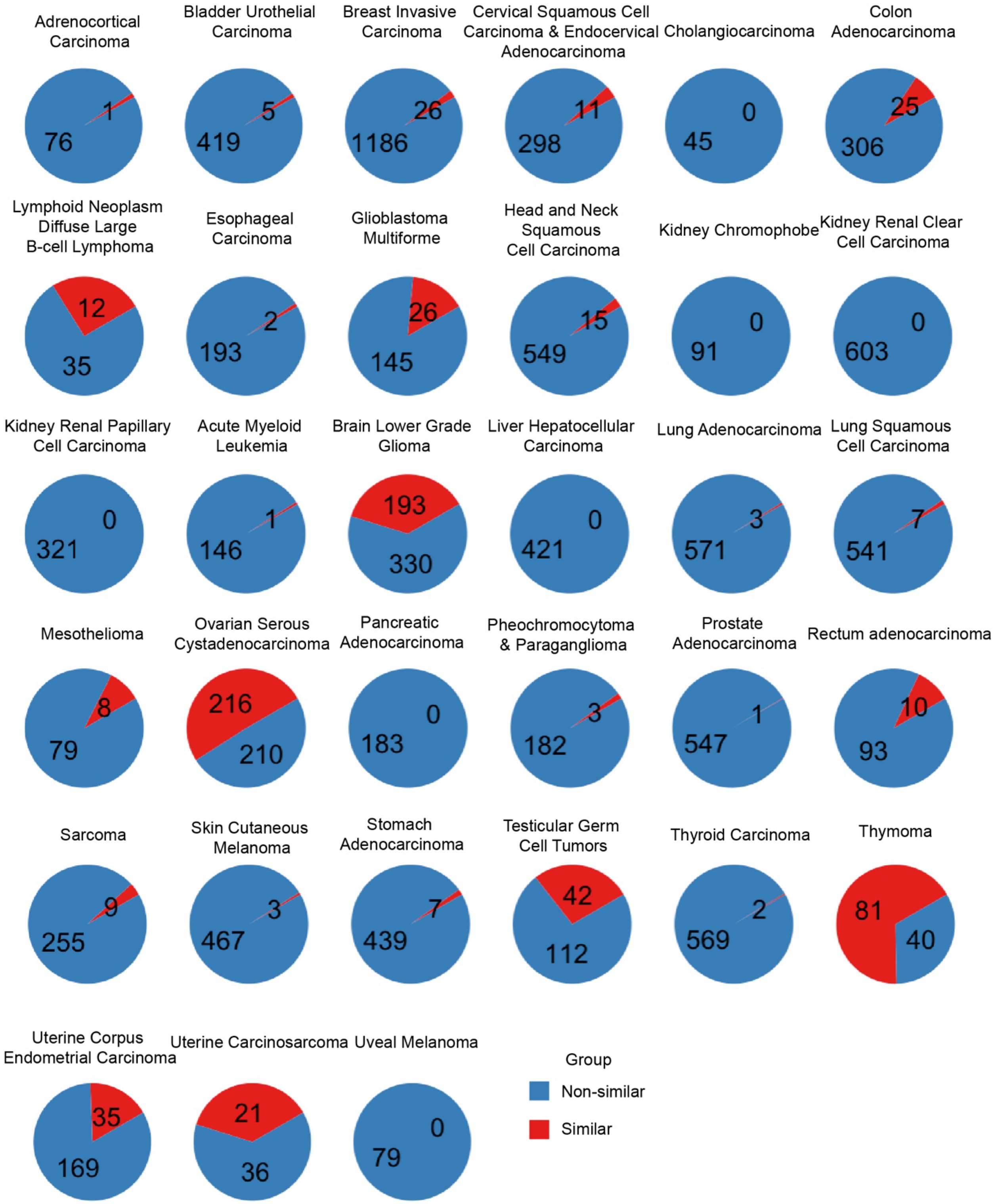

Fig. S4. Plot of numbers of human tumor samples by cancer types with similar metabolic signatures to the fly *scrib* mutant tumors.

Disease Models & Mechanisms • Supplementary information

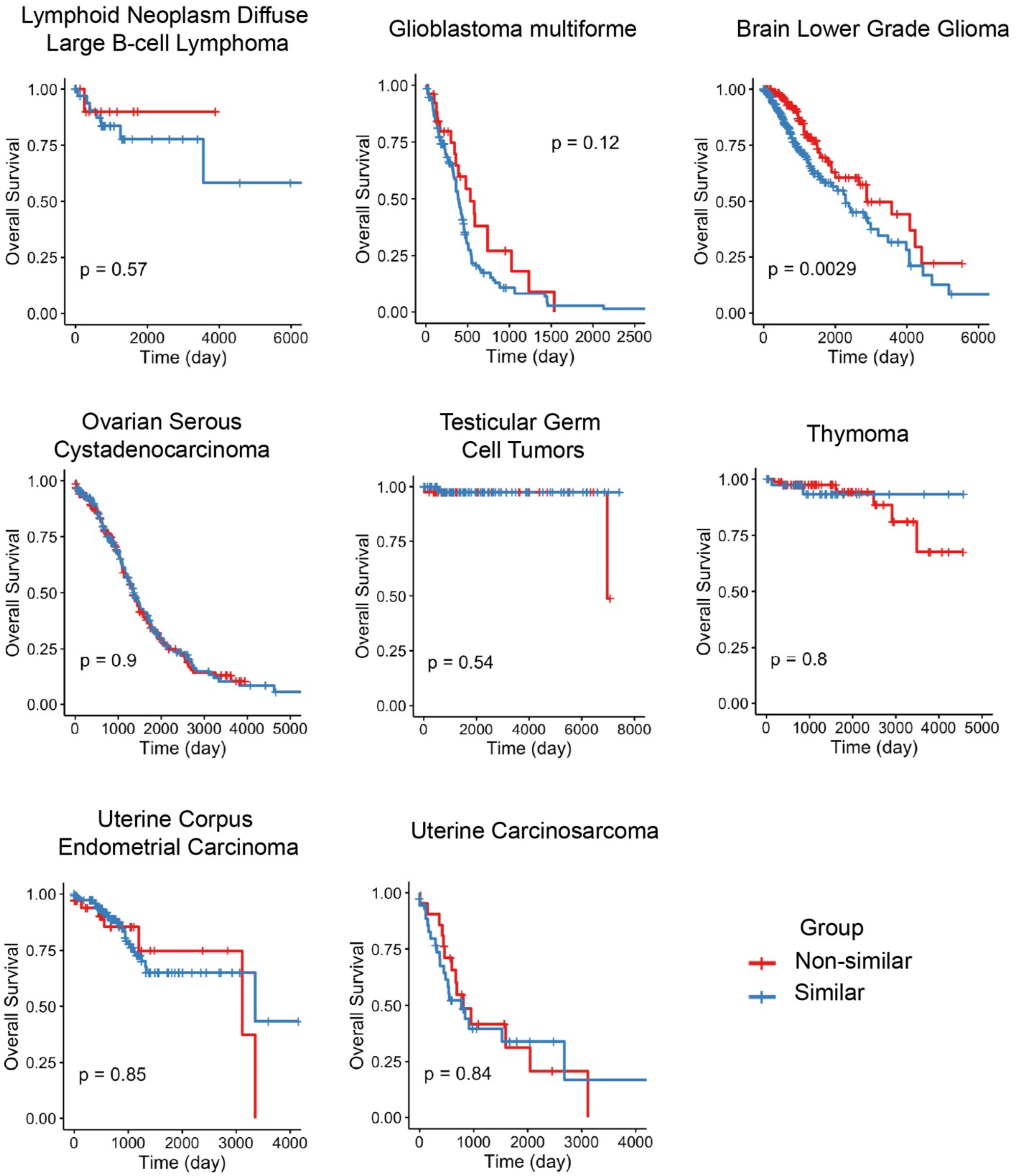

Fig. S5. Plot of patient survival curves by cancer types comparing human tumors with high metabolic similarity to fly tumors and the non-similar group.

P-values are calculated by log-rank test and represent whether there is a significant difference on survival between metabolically similar and non-similar groups for each human cancer type.

Disease Models & Mechanisms • Supplementary information

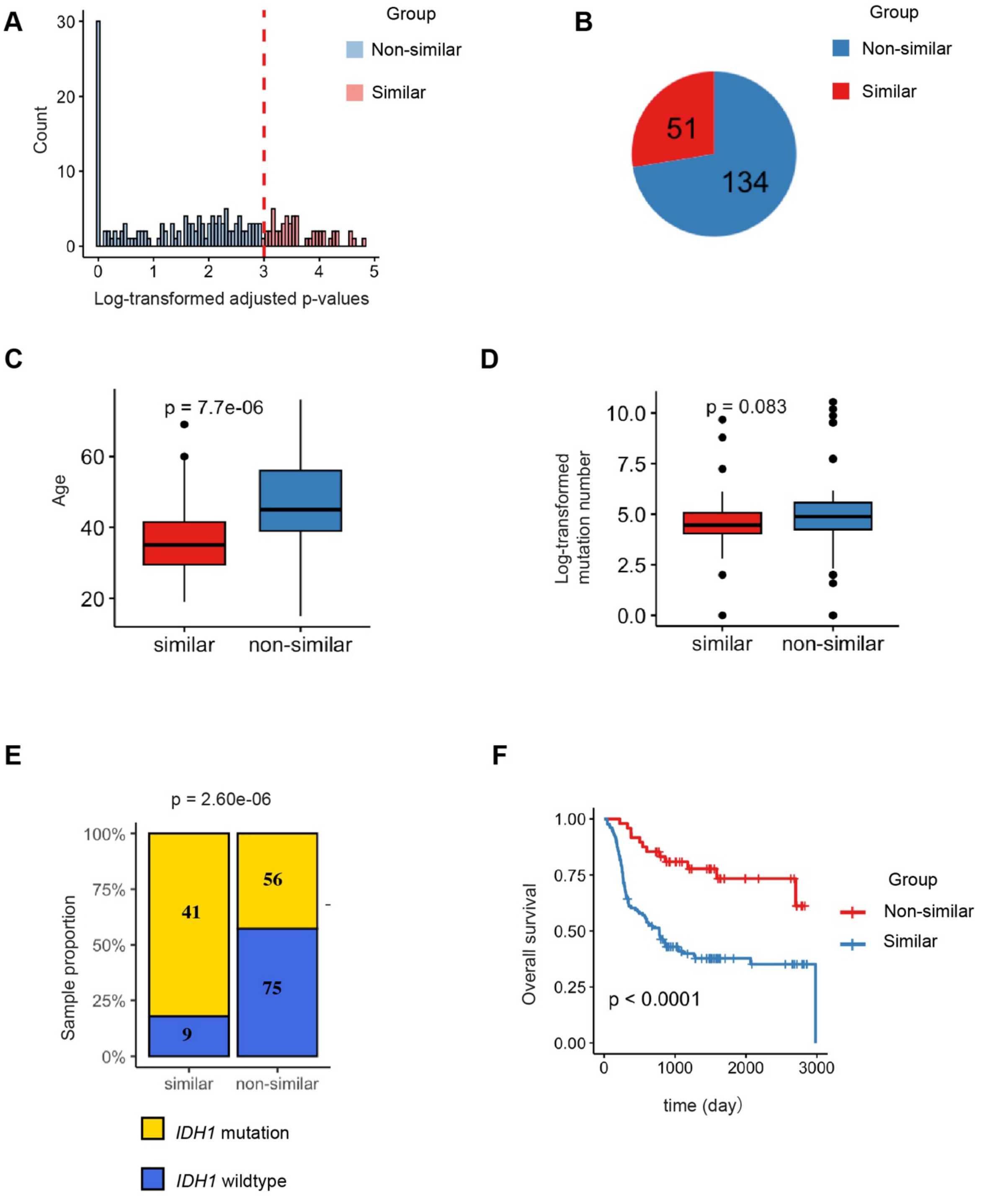

Fig. S6. Analysis of 185 human glioma samples from CGGA database.

(A) Plot of the distribution of adjusted p-values for metabolic similarity tests between human glioma samples from CGGA dataset and the fly *scrib* mutant tumors. P-values are calculated by Pearson correlation test and human tumor samples with p-values lower than

1e-03 (colored in red) are recognized as samples with significantly similar metabolic patterns with fly.

(B) 51 out of 185 human glioma samples exhibit metabolic signatures similar to those of the fly *scrib* mutant tumors based on p-values of Pearson correlation test.

(C) Patients with glioma metabolically similar to the fly tumors are significantly younger than others. Data represents distribution of patient age of two human tumor groups from CGGA and p-value is calculated by t-test.

(D) Human glioma samples metabolically similar to the fly tumors acquire a lower number of mutations than other samples. Data represents log2-transformed count number of mutation events detected in two human tumor groups from CGGA and p-value is calculated by t-test.

(E) Human glioma samples metabolically similar to the fly tumors show an enrichment of *IDH1* mutation. Numbers in the bar plot shows the number of patients with or without *IDH1* mutation in the two human tumor groups. P-value is calculated by Fisher's exact test which represents significant enrichment of *IDH1* mutation in the metabolically similar tumor group.

(F) Patients with glioma metabolically similar to the fly tumors have better survival outcomes than the non-similar group. P-value is calculated by log-rank test and represents significantly different survival between two human tumor groups.

Table S1. List of metabolite concentrations from the control and the *dlg* mutant larvae as measured in the 600MRM metabolite analysis with LC-MS.

Available for download at
https://journals.biologists.com/dmm/article-lookup/doi/10.1242/dmm.052313#supplementary-data

Table S2. The expression of transposons in the *scrib* mutant tumors over time.

Available for download at
https://journals.biologists.com/dmm/article-lookup/doi/10.1242/dmm.052313#supplementary-data

Table S3. The transposition events mapped in the *scrib* mutant tumors over time in comparison with the reference genome.

Available for download at
https://journals.biologists.com/dmm/article-lookup/doi/10.1242/dmm.052313#supplementary-data

Table S4. List of conserved metabolic genes and pathways in *Drosophila Melanogaster* and human.

Available for download at
https://journals.biologists.com/dmm/article-lookup/doi/10.1242/dmm.052313#supplementary-data
